# Oxidative stress and mitochondrial dysfunction in Kindler syndrome

**DOI:** 10.1186/s13023-014-0211-8

**Published:** 2014-12-21

**Authors:** Elisabeth Zapatero-Solana, Jose Luis García-Giménez, Sara Guerrero-Aspizua, Marta García, Agustí Toll, Eulalia Baselga, Maria Durán-Moreno, Jelena Markovic, Jose Manuel García-Verdugo, Claudio J Conti, Cristina Has, Fernando Larcher, Federico V Pallardó, Marcela Del Rio

**Affiliations:** Centre for Biomedical Network Research on Rare Diseases (CIBERER), ISCIII, Valencia, Spain; Regenerative Medicine Unit. Departament of Basic Research, Centro de Investigaciones Energéticas, Medioambientales y Tecnológicas (CIEMAT), Madrid, Spain; Department of Bioengineering, Universidad Carlos III de Madrid (UC3M), Madrid, Spain; Instituto de Investigación Sanitaria de la Fundación Jiménez Díaz (IIS-FJD), Madrid, Spain; Department of Physiology, Faculty of Medicine, University of Valencia, Valencia, Spain; Fundación Investigación Hospital Clínico Universitario de Valencia, Instituto de Investigación INCLIVA, Valencia, Spain; Servei de Dermatologia, Hospital del Mar, Parc de Salut Mar, Cancer Research Program, IMIM (Institut Hospital del Mar d’Investigacions Mèdiques), Barcelona, Spain; Department of Dermatology, Hospital de la Santa Creu i Sant Pau, Barcelona, Spain; Laboratorio de Neurobiología Comparada, Instituto Cavanilles, Universidad de Valencia, CIBERNED, Valencia, Spain; Department of Dermatology, Medical Centre-University of Freiburg, Freiburg, Germany

**Keywords:** Kindlin1, Oxidative stress, Mitochondria, Keratinocytes, Genodermatosis

## Abstract

**Background:**

Kindler Syndrome (KS) is an autosomal recessive skin disorder characterized by skin blistering, photosensitivity, premature aging, and propensity to skin cancer. In spite of the knowledge underlying cause of this disease involving mutations of *FERMT1 (fermitin family member 1)*, and efforts to characterize genotype-phenotype correlations, the clinical variability of this genodermatosis is still poorly understood. In addition, several pathognomonic features of KS, not related to skin fragility such as aging, inflammation and cancer predisposition have been strongly associated with oxidative stress. Alterations of the cellular redox status have not been previously studied in KS. Here we explored the role of oxidative stress in the pathogenesis of this rare cutaneous disease.

**Methods:**

Patient-derived keratinocytes and their respective controls were cultured and classified according to their different mutations by PCR and western blot, the oxidative stress biomarkers were analyzed by spectrophotometry and qPCR and additionally redox biosensors experiments were also performed. The mitochondrial structure and functionality were analyzed by confocal microscopy and electron microscopy.

**Results:**

Patient-derived keratinocytes showed altered levels of several oxidative stress biomarkers including MDA (malondialdehyde), GSSG/GSH ratio (oxidized and reduced glutathione) and GCL (gamma-glutamyl cysteine ligase) subunits. Electron microscopy analysis of both, KS skin biopsies and keratinocytes showed marked morphological mitochondrial abnormalities. Consistently, confocal microscopy studies of mitochondrial fluorescent probes confirmed the mitochondrial derangement. Imbalance of oxidative stress biomarkers together with abnormalities in the mitochondrial network and function are consistent with a pro-oxidant state.

**Conclusions:**

This is the first study to describe mitochondrial dysfunction and oxidative stress involvement in KS.

**Electronic supplementary material:**

The online version of this article (doi:10.1186/s13023-014-0211-8) contains supplementary material, which is available to authorized users.

## Background

Kindler Syndrome (KS; OMIM 173650; ORPHA 2908), a rare heritable skin disorder with a complex phenotype and poorly understood pathogenesis, is characterized clinically by acral skin blisters in infancy and childhood, photosensitivity, and progressive poikiloderma [1-3]. Additional clinical features include chronic gingival erosions, oesophageal and urethral stenosis as well as high risk of mucocutaneous malignancies [1].

KS results from recessive loss-of-function mutations in the *FERMT1* (fermitin family member 1) gene that encodes the protein kindlin-1, a component of focal adhesions in epithelial cells [2,3]. This protein mediates anchorage between the actin cytoskeleton and the extracellular matrix via focal adhesions, playing an important role in keratinocyte migration, proliferation and adhesion [4-7].

Although genetic mutations in *FERMT1* have been identified as the origin of this disease, the complex phenotype of KS cannot be exclusively explained based on the adhesive function of kindlin-1. Therefore, the mechanisms responsible for clinical features such as photosensitivity and cancer are still awaiting to be unveiled [8]. Several pathognomonic features of KS, not related to skin fragility such as aging, inflammation and cancer have been strongly associated to oxidative stress [9]. Reactive oxygen species (ROS) are produced continuously in tissues as part of normal cell functions. However, the excessive production of ROS induces DNA and other macromolecules damage [9-11]. To counteract the excessive production of ROS, mammalian cells have developed several mechanisms of detoxification, located in specific subcellular compartments [12]. These include non-enzymatic antioxidants such as glutathione (GSH) and enzymes with antioxidant properties (e.g. catalase and superoxide dismutases) [9,13]. Glutathione is one of the main antioxidant molecules with a role in ROS detoxification and the biochemical systems involved in their synthesis and recovery (glutathione reductase and glutathione peroxidase) are important to maintain the cell in a physiologic redox status [14,15].

In this study we sought to analyse at both, cellular and molecular levels, the potential derangements of the redox status in KS skin and keratinocytes. Using a variety of biochemical, molecular and morphological approaches we were able to detect an imbalance of oxidative stress biomarkers and mitochondrial abnormalities consistent with a pro-oxidant state in KS. Our results provide pathological bases for the non-adhesive clinical manifestations of this intriguing genodermatosis.

## Methods

### Skin biopsies

Skin biopsies were taken from non-affected areas of KS patients’ arm, in which mutations, ages and gender are detailed in (Additional file 1: Table S1). Patient informed consents were obtained in agreement with the collaborative centres, where biopsies and blood samples were obtained. The Ethics Committee of Fundación Jiménez Díaz (Madrid, Spain) evaluated and approved this research, stating that the procedures followed were in accordance with the institutional ethical standards on human experimentation and the project adheres to the Helsinki Guidelines and further reviews including Seul 2008.

### Electron microscopy

For Electron Microscopy, cell cultures were fixed with 3.5% glutaraldehyde while biopsies were fixed with 2% paraformaldehyde and 2.5% glutaraldehyde solution by immersion. All samples were post-fixed in 2% osmium and dehydrated through an ascending series of ethanol concentrations. They were then stained with 2% uranyl acetate in 70% ethanol for 2 hours and embedded in Durcupan resin (Fluka BioChemika, Ronkokoma, NY, USA). Ultrathin sections (70 nm) were cut, stained with Reynolds’ lead citrate and examined under a Transmission Electron Microscope (FEI Tecnai G2 Spirit, FEI Europe, Eindhoven, Netherlands) using a digital camera (Morada, Soft Imaging System, Olympus, Japan). To identify the ultrastructural differences between patients’ skin specimens and controls, 10 randomized cells from 10 randomized areas of each cell culture were analyzed. Skin biopsies were obtained from control and KS patients and ten randomized mitochondria from eight keratinocytes were analyzed for each sample.

#### Mutational analysis

Intronic primer pairs were designed to amplify individual exons and flanking splice sites of the *FERMT1* gene. Polymerase Chain Reaction (PCR) amplification of *FERMT1* gene was performed on genomic DNA as previously described [2,16]. PCR products were directly sequenced in both orientations in an ABI Prism 3730 genetic analyzer (Life Technologies/Applied Biosystems).

### Primary keratinocyte culture

Skin biopsies were incubated two hours at room temperature with collagenase (Sigma) (0.25% diluted in DMEM (Gibco, Life Technologies)). Detached epidermal sheet was then incubated with tryspin solution (Sigma) for 20 minutes at 37°C (four cycles of trypsin were done). The released keratinocytes were centrifuged at 1000 rpm for 7 minutes [17,18]. The cell pellet was resuspended in keratinocyte medium: 3:1 mixture of Dulbecco’s modified Eagle medium (DMEM) (GIBCO-BRL) and HAM’s F12 (Gibco, Life Technologies), containing 10% fetal calf serum replacement (Fetal Clone II, Hyclone-Lonza). This medium was supplemented as previously described [19,20]. Keratinocytes were plated in T75 flasks previously seeded with a feeder layer of lethally irradiated (X-ray; 50 Gy) 3 T3-J2 cells (a gift from Dr J. Garlick, SUNY, Stony Brook, NY) as previously described [21], for western blot and redox biosensors experiments. In contrast, for oxidative stress markers, confocal microscopy and electron microscopy experiments cells were cultured in Cnt-BM.1 Basal Medium (CellNTec) in feeder layer free conditions. Cells were cultured at 37°C in a humid atmosphere containing 5% CO_2_ and the culture medium was changed every other day. Third to fifth passages cells were used as indicated for all experiments.

### Measurement of lipid peroxides

Lipid peroxides were determined by measuring MDA, which is formed from such peroxides. MDA from the samples reacted with thiobarbituric acid (TBA) at 100°C to form a MDA-TBA adduct. The protein-free extract was separated by HPLC (Ultimate 3000 Bionex) on a column of octadecyl silica gel (C16, Bionex) to separate the MDA-TBA adduct from interfering chromogens. The adduct was eluted from the column with 50 mM phosphate buffer pH 6.8- methanol 50% and quantified spectrophotometrically at 532 nm.

### Measurement of GSSG/GSH ratio

GSH and GSSG levels were studied using the Glutathione Fluorescent Detection Kit (Arbor Assays, Ann Arbor, Michigan USA) following the instructions of manufacturers for cellular material. Briefly, cells were prepared in 5% sulphosalicilic acid and centrifuged at 13,000 g to separate the proteins. The supernatant containing the GSH and GSSG was reacted with ThioStarreagent to produce a fluorescent product (λ emission 510 nm, λ excitation 390 nm). Addition to the sample of a reaction mixture containing NADPH and GSH reductase converts all the GSSG into free GSH, which then reacts with ThioStar, yielding the signal corresponding to total GSH. The difference between both measures offers the amount of GSSG.

### qPCR

Total RNA was isolated from cells using the PARISTM Protein and RNA Isolation System (Ambion; Austin, TX) according to the manufacturer’s instructions. For reverse transcription reactions (RT), 1 μg of the purified RNA was reverse transcribed using random hexamers with the High-Capacity cDNA Archive kit (Applied Biosystems, Foster City, CA) according to the manufacturer’s instructions. RT conditions comprised an initial incubation step at 25°C for 10 min to allow random hexamers annealing, followed by cDNA synthesis at 37°C for 120 min, and a final inactivation step for 5 min at 95°C. The mRNA levels were determined by quantitative real-time PCR analysis using an ABI Prism 7900 HT Fast Real-Time PCR System (Applied Biosystems, Foster City, CA). Gene-specific primer pairs and probes for GCLC and GCLM (GCLM:Hs00157694_m1, GCLC:Hs00155249_m1,Assay-on-demand, Applied Biosystems), were used together with 1x TaqMan® Universal PCR Master Mix (Applied Biosystems, Foster City, CA) and 2 μL of reverse transcribed sample RNA in 20 μL reaction volumes. PCR conditions were 10 min at 95°C for enzyme activation, followed by 40 two-step cycles (15 sec at 95°C; 1 min at 60°C). The levels of glyceraldehyde-3-phosphate dehydrogenase (GAPDH) expression were measured in all samples to normalize gene expression for sample-to-sample differences in RNA input, RNA quality and reverse transcription efficiency. Each sample was analyzed in triplicate, and the expression was calculated according to the 2-ΔΔCt method [22].

### Redox biosensors experiments

Glutaredoxin-1 (Grx1) is an enzyme that specifically catalyzes the equilibrium between the redox pair of interest, reduced glutathione (GSH) and oxidized glutathione (GSSG) in the cytoplasm. In this way, Grx1-roGFP2 fusion protein allowed dynamic live imaging of the glutathione redox potential in the cytoplasm with high sensitivity (from millimolar to nanomolar changes) and temporal resolution, facilitating the observation of physiologically relevant redox-based signals responding to exogenously applied agents [23,24]. The Grx1-roGFP2 fusion protein targeted to the mitochondrial matrix using a signal sequence from *Neurospora crassa* ATP synthase protein 9 (mito-Grx1-roGFP2) determines the glutathione redox potential in mitochondria.

Retroviral supernatants from PA317 Grx1-roGFP2 and mito-Grx1-roGFP2 lines (kindly donated by Dr. Santiago Lamas, CIB) were collected and used for control and patients human keratinocytes infections (three controls and three patients). Two rounds of seven hours infections were performed and keratinocytes were grown until confluence with keratinocyte medium (percentage of infection was higher than 75%). GFP positive cells were analyzed by flow cytometry (LSRFortessa, BD Biosciences, USA) either in basal state or after addition of 12.5 μM hydrogen peroxide solution. Cells were excited with 405 and 488 nm lasers and the ratio of emissions in the green channel was calculated. Flow cytometry data were analysed using FlowJo version 7.6.1.

### Western blotting

Keratinocytes were lysed with a lysis buffer prepared with Tris pH 7.5 50 mM, NaCl 150 mM, Triton 1.5%, EDTA 1 mM, protease inhibitor cocktail tablets (Roche), ortovanadate, sodium pyrophosphate and sodium fluoride. The lysates were then loaded with LDS Sample Buffer (Invitrogen) or manufactured sample buffer 5X and run on NuPage 4–12% Bis-Tris gels (Invitrogen) at 120 V for 2 hours for antioxidant enzymes. The proteins were then transferred onto nitrocellulose membranes (Invitrogen) and blocked in 5% skimmed milk in 0.1% Tween-20 (Sigma-Aldrich) and phosphate-buffered saline. The membranes were then probed with anti-kindlin1 (1:10.000) antibody [25] overnight at 4°C. Mouse monoclonal anti-α tubulin (Sigma-Aldrich) was used as loading control. Anti-rabbit horseradish peroxidase-conjugated IgG antibody was used as secondary antibody. Visualization of protein bands was done with ECL western blotting detection reagents (Amersham Biosciences and Thermo Scientific).

### Confocal microscopy

The mitochondrial distribution and morphology was studied by confocal microscopy. Cells were plated in rounded glass in Cnt-BM.1 medium and after 48 h of culture they were stained to localize mitochondria and nuclei. Cells were loaded with Mito Tracker Red™ (Invitrogen) in a final concentration of 250 nM in cell culture medium, 37°C and 5% CO2 and after 30 minutes of incubation they were mounted with DAPI (Molecular probes). The fluorescence was detected by confocal microscopy (A1 Confocal Laser Microscope System (Nikon)) and the plane (along the Z axes) that had most mitochondria was captured. Images are representative examples of 3 separate experiments.

Mitocondrial’s membrane potential was determined with JC-1™ vital dye (Invitrogen). It is a cationic dye that exhibits potential-dependent accumulation in mitochondria, indicated by a fluorescence emission shift from green (~520 nm) to red (~590 nm). Consequently, mitochondrial depolarization is indicated by a decrease in the red/green fluorescence intensity ratio. Cells were plated in rounded glass in Cnt-BM.1 medium and after 48 h of culture they were stained with 2.5 μg/mL JC-1™, 10 minutes, 37°C. After that, glasses were mounted with DAPI and pictures were obtained with a confocal microscope.

### Statistical analysis

For the statistical analysis of the results, the mean was taken as the measurement of the main tendency, while standard deviation was taken as the dispersion measurement. A Student-Newman-Keuls method was used to determine the significance of differences, when analyzing GSSG/GSH ratio, GCLC and GCLM relative expression determined by qRT-PCR, MDA levels studied by HPLC-UV, biosensors by flow cytometry and JC-1 intensity by confocal microscopy. The significance has been considered at *p < 0.05, **p < 0.01 and ***p < 0.001, as indicated in each case. GraphPad Software v5.0 was used for statistical analysis and graphic representations.

## Results

### FERMT1 mutations correlate with reduced or absent kindlin-1 protein in KS keratinocytes

Three unrelated patients with KS were studied. The patients’ mutations, age and gender are summarized in Additional file 1: Table S1. Clinical features and mutations have been previously described; patients 4, 10 and 53 described in that study correspond respectively to our patients SK1, SK2 and SK3 [16]. The effects of the mutations were confirmed at mRNA and protein levels. Patient 1 (SK1) presented a homozygous mutation within the consensus sequence of the donor splice site of intron 11 (c.1371 + 4A > G) that was predicted to result in aberrant splicing of the FERMT1 pre-mRNA. This mutation causes the skipping of 32 nucleotides leading to incorrect processing of the mRNA that generates a prematurely ending (truncated) protein. Consistently, the RT-PCR analysis and the western blot indicated the presence of truncated mRNA (data not shown) and protein (p.Gln226ProfsX17) (Additional file 1: Figure S1). Patient 2 (SK2) presented a homozygous duplication at codon 676 (c.676dupC) leading to a frameshift that results in the generation of a premature termination codon, 16 codons downstream. Consistent with this prediction the mRNA and protein analysis showed the presence of a truncated message (data not shown) and absence of protein (Additional file 1: Figure S1). Patient 3 (SK3) presented a homozygous single nucleotide substitution (T > C) at position 1198 in exon 10 (c.1198 T > C). mRNA and protein analysis showed messenger (data not shown) and protein of normal size (amino acid substitution: p.Ser400Pro [16]), although the amount of kindlin-1 is markedly reduced as compared to control keratinocytes (Additional file 1: Figure S1).

These patients represent a proper sampling of the spectra of mutations found in KS, with one patient showing total lack of expression (SK2), one presenting a truncated protein (SK1) and a third expressing a normal size protein with a single amino acid substitution and reduced expression (SK3).

### Keratinocytes from KS patients are prone to oxidative stress

**Figure 1:**
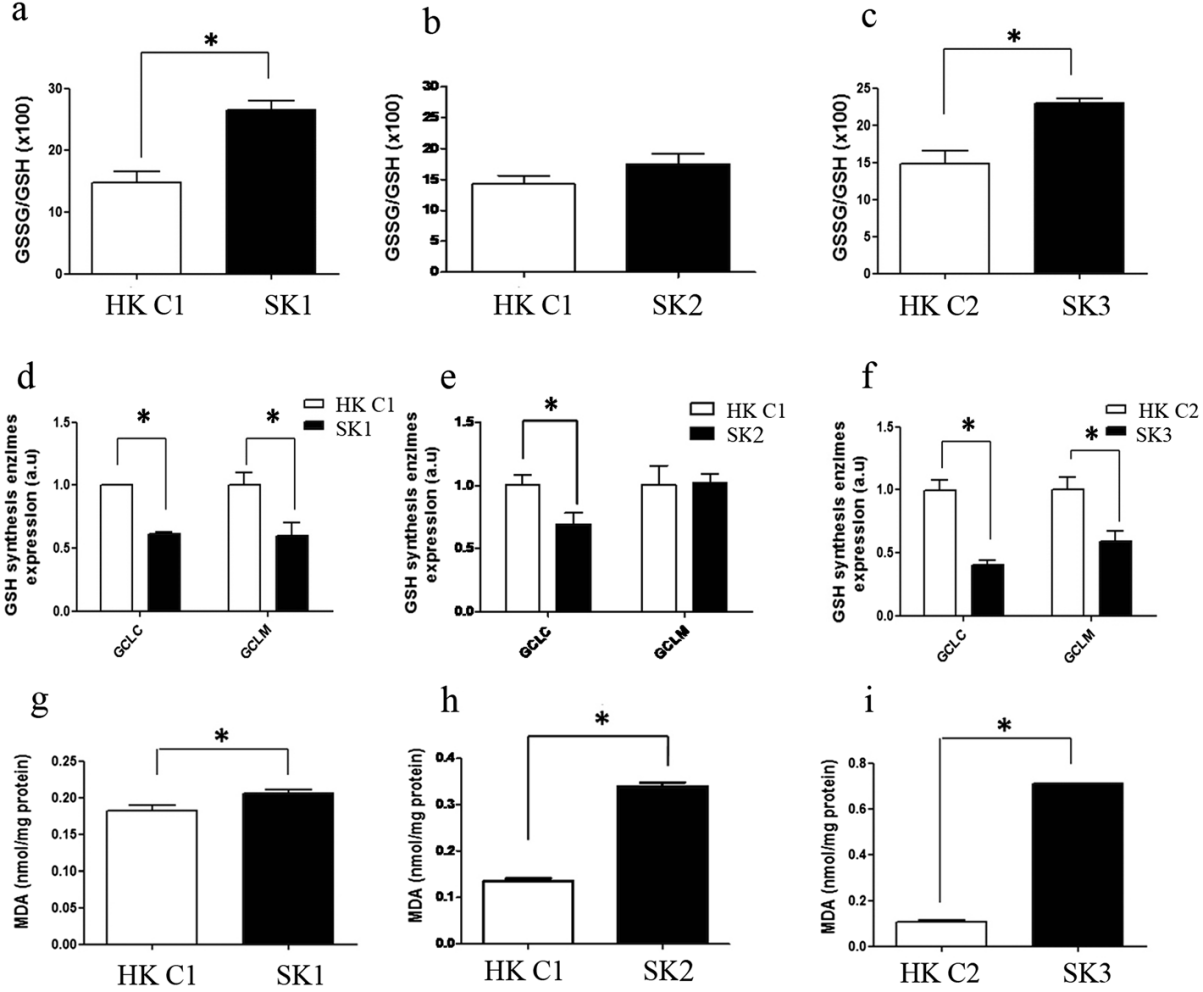
**Oxidative stress profile in Kindler Syndrome Keratinocytes. (a-c)** GSSG/GSH ratio as redox profile coefficient was measured by fluorimetry in keratinocytes from patients SK1 **(a)**, SK2 **(b)**, and SK3 **(c)** and their matched controls. **(d-f)** GCLC and GCLM gene expression was analyzed by qPCR using GAPDH as reference gene in SK1 **(d)**, SK2 **(e)**, and SK3 **(f)** and their matched controls, using the method 2^-ΔΔCt^. **(g-i)** Malondialdehyde (MDA) levels were measured by HPLC-UV in SK1 **(g)**, SK2 **(h)**, and SK3 **(i)** and their matched controls. Results represent the means and SD from two independent experiments in triplicate samples. *p < 0.05: statistically significant difference from control value, after *t-*student test.

GSH is one of the most important protective mechanisms against ROS. In this regard, one of the best-characterized biomarkers of cellular oxidative stress is the ratio between oxidized and reduced glutathione (GSSG/GSH) [13-15]. The GSSG/GSH ratio was significantly higher in keratinocytes from SK1 and SK3 patients than those obtained from their matched (gender and age) healthy controls. Keratinocytes from SK2 patient followed the same trend, although differences were not statistically significant (Figure 1a-c). Expression levels of the two subunits of the gamma-glutamyl cysteine ligase (GCLC, the catalytic subunit and GCLM, the modulatory subunit), which catalyzes the first rate limiting step for the synthesis of GSH were determined by quantitative PCR. Results showed in Figure 1d-f, indicated that the mRNA levels of GCLC were significantly reduced in KS keratinocytes, as compared to their matched controls. Decreased levels of the modulatory subunit (GCLM) mRNA were also found in SK1 and SK3.

We also studied another marker of oxidative stress, malondialdehyde (MDA). This is a product of the degradation of polyunsaturated lipids by ROS [26]. Our results showed that all KS patient-derived keratinocytes have higher MDA levels compared to their matched control keratinocytes, indicating oxidative damage of lipids (lipoperoxidation) in KS cells. Noteworthy, MDA level values were higher in cells obtained from an adult patient (SK3) than in the keratinocytes derived from the young patients (SK1 and SK2) (Figure 1g-i).

**Figure 2:**
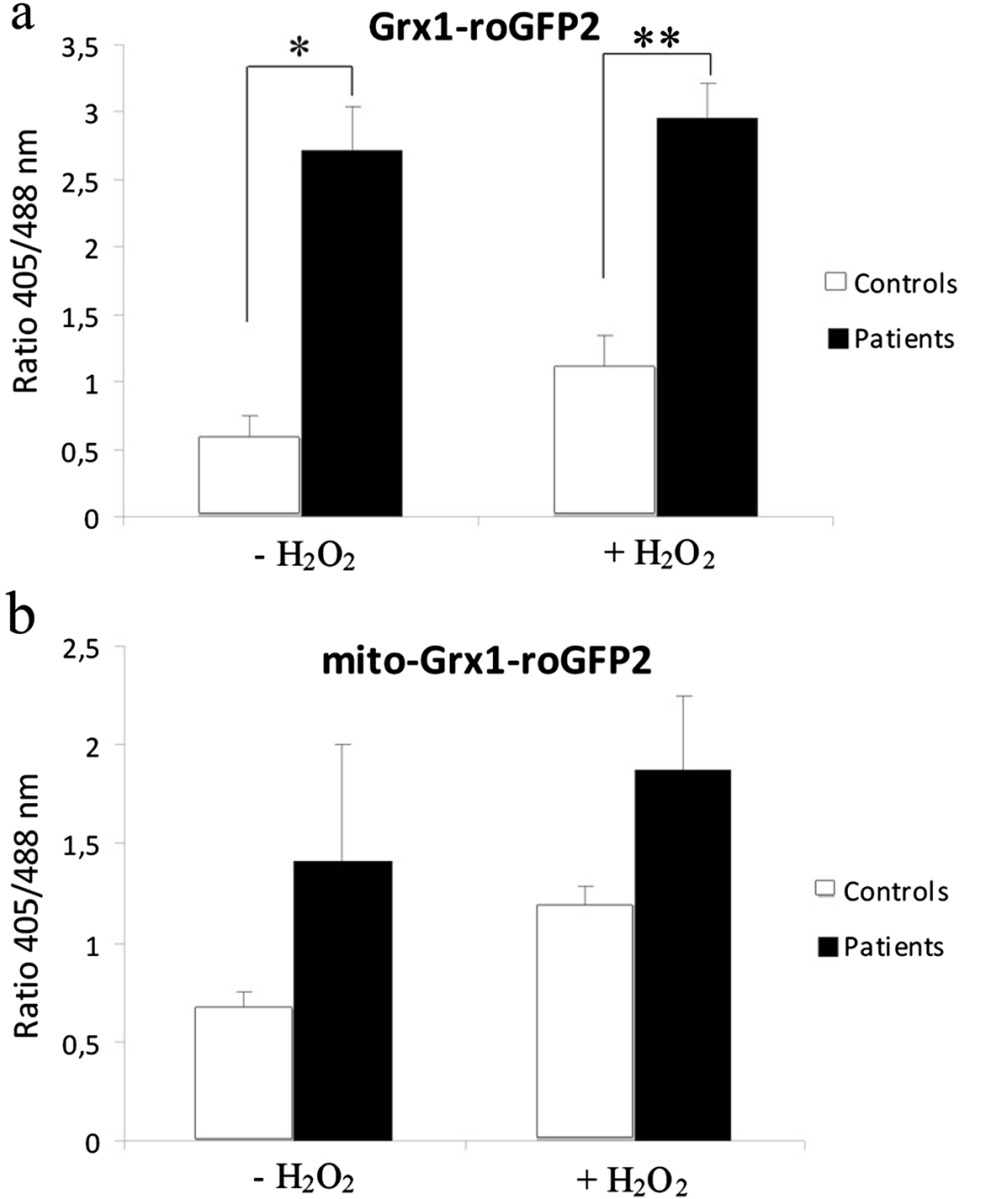
**Redox biosensor experiments. (a and b)** Retroviruses encoding either Grx1-roGFP2 **(a)** or mito-Grx1-roGFP2 **(b)** cDNAs were used to infect KS patients and control keratinocytes. The Ratio 405/488 nm was calculated in basal state and when H_2_O_2_ solution was added to the cells (12.5uM). KS patients present higher ratios than controls even at a basal state (without H_2_O_2_ solution), indicating a higher oxidized state in both cytoplasmic and mitochondria compartments. Average values of each experimental group are shown. *p < 0.05, **p < 0.01: statistically significant difference from control value, after *t-*student test.

In order to confirm the redox status alteration in KS keratinocytes were transduced with a highly sensitive chimerical redox biosensor system (Grx1-roGFP2). A second sensor containing a signal peptide directed to the mitochondria (mito-Grx1-roGFP2) was also used to detect oxidative stress in the mitochondrial matrix*.* KS and control keratinocytes expressing Grx1-roGFP2 or mito-Grx1-GFP2 were treated with hydrogen peroxide solution (H_2_O_2_) and analyzed by flow cytometry. The basal biosensor levels in the KS keratinocytes (in the absence of H_2_O_2_) already showed a higher oxidized/reduced ratio than their respective controls. After H_2_O_2_ challenge, the same tendency was observed, indicating a higher pro-oxidative state in KS keratinocytes (Figure 2a). When the mitochondrial redox status was analyzed using mito-Grx1-roGFP2, a similar response to that found at the cytoplasm was observed (Figure 2b). Only patient 3 showed similar responses to the controls (Additional file 1: Figure S2). This could be likely due to the mild nature of the *FERMT1* mutation in this patient which explains also the moderate symptoms described in this patient.

### Mitochondrial structure, localization and function is altered in KS epidermis and keratinocytes in culture

**Figure 3:**
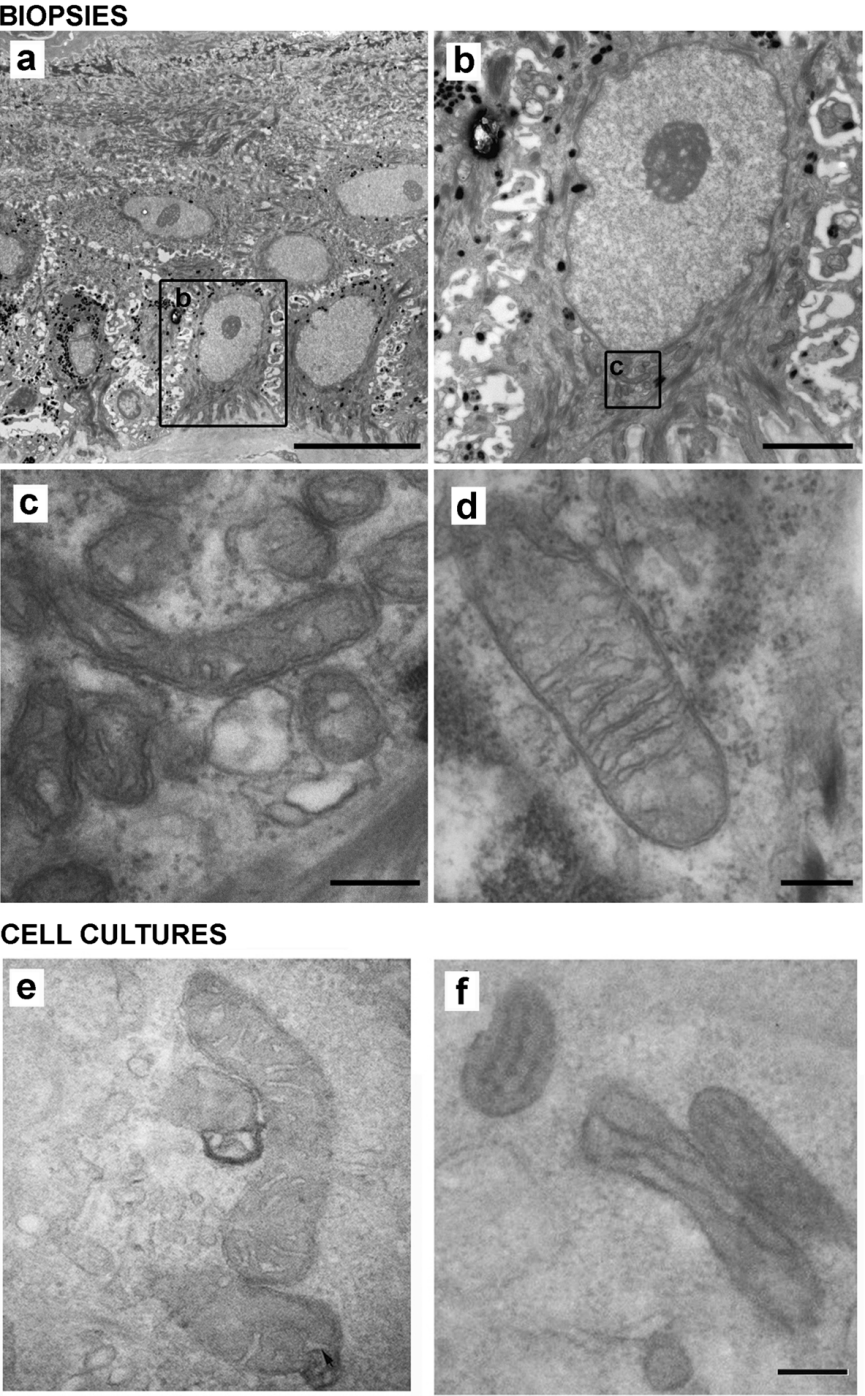
**Mitochondrial ultrastructure in KS.** Keratinocytes from skin biopsies and cultured keratinocytes from control and KS patients were studied by electron microscopy (EM). **(a)** Panoramic view of the basal stratum from SK3 patient. **(b)** Detail of a keratinocyte (squared region in **a**). **(c)** Mitochondria from patient SK3 (squared in **b**). **(d)** Mitochondria from a control sample. **(e,f)** EM photomicrographs of mitochondria from control **(e)** and from patient SK1 **(f)** cultured keratinocytes. Scale bars: 10 μm **(a)**, 2 μm **(b)**, 200 nm **(c, d, e, f)**. Images shown are representative of each experimental group.

**Figure 4:**
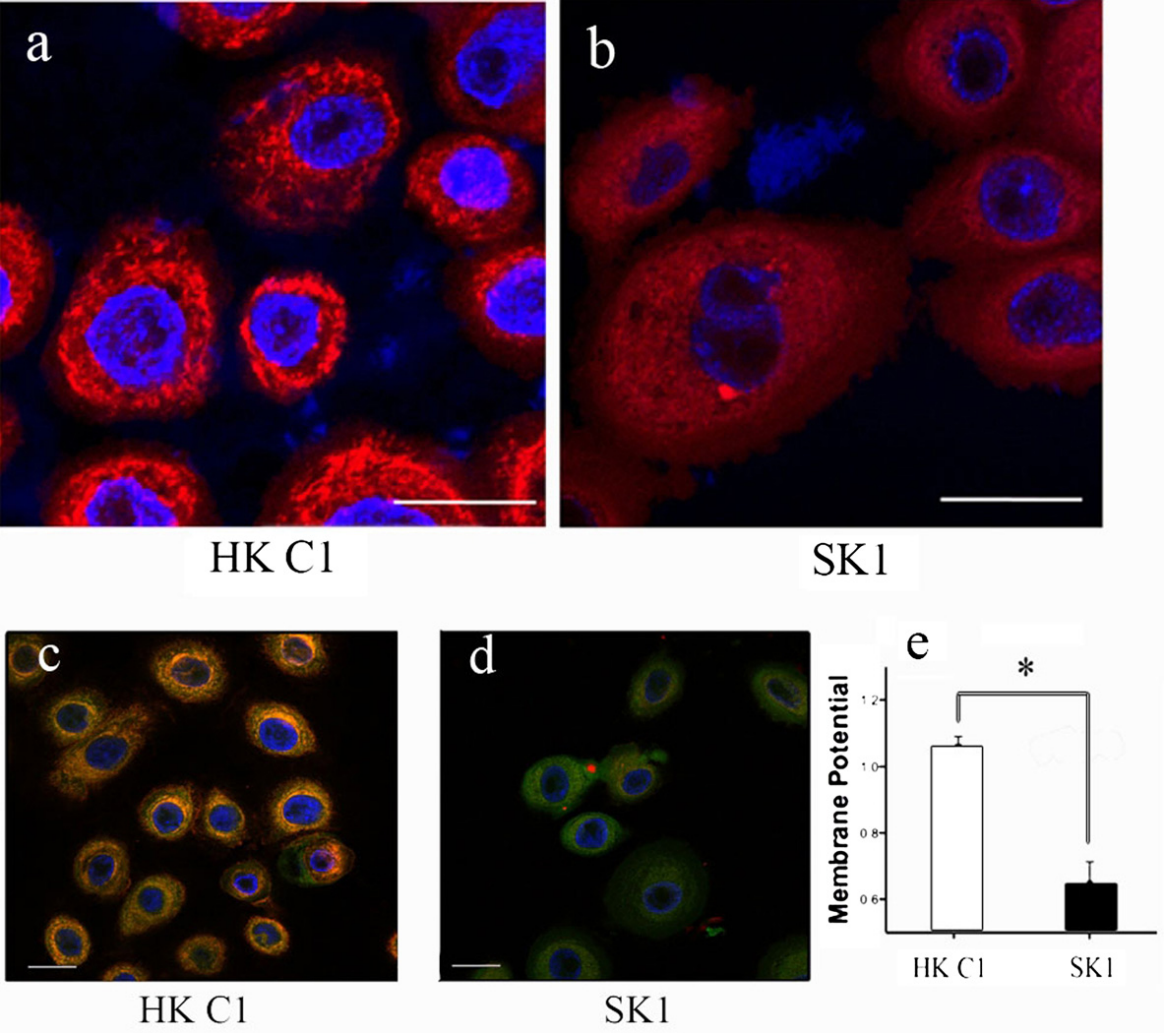
**Mitochondria distribution and function in KS keratinocytes. (a and b)** Mito Tracker Red staining. Note the smeared mitochondria staining in KS keratinocytes **(b)** as compared to the control cells **(a). (c and d)** JC-1 staining. Note mitochondrial depolarization in KS keratinocytes as indicated by the decrease in the red/green fluorescence intensity ratio. **(e)** Quantification of JC-1 staining. Membrane potential reduction was statistically significant (*p < 0.05) after *t-*student test. Scale bars = 10 μm.

We sought to determine whether deranged redox status could translate into ultrastructural changes in target organelles. For this purpose, the morphology of mitochondria in patient skin biopsies and cultured keratinocytes was analyzed by electron microscopy. The Figure 3a shows a panoramic view of the KS epidermis, showing a zoom in a basal keratinocyte (Figure 3b). The ultrastructural analysis of KS skin biopsies revealed striking abnormalities in mitochondria. In fact, mitochondrial crests were irregular, dilated and did not present parallel distribution (Figure 3c), which contrasted with the well-organized mitochondrial network in normal skin biopsies (Figure 3d). Furthermore, both internal and external mitochondrial membranes had a wavy morphology and irregular thickness (Figure 3c). Similarly, the ultrastructural analysis of the cultured keratinocytes from KS patients showed mitochondrial alterations with a tendency toward the fusion of mitochondrial crests that may affect the inter-membrane space and functionality (Figure 3f) compared with control keratinocytes (Figure 3e). To analyze mitochondrial distribution, keratinocytes from healthy subjects and KS patients were incubated with the mitochondria-specific dye Mito Tracker Red and subsequently analyzed by confocal microscopy. Mitochondria in control keratinocytes formed a well-established network. In contrast, keratinocytes from KS patients showed both, a reduced and diffuse Mito Tracker Red staining consistent with a disorganized mitochondrial network (Figure 4a and b). In order to assess mitochondrial function, the membrane potential was studied in control and KS keratinocytes using the JC-1 probe. The analysis showed a significant reduction of the membrane potential in KS cells compared to controls as determined by the red-to-green JC-1 dye shift (Figure 4c-e and Additional file 2: Figure S3). Overall, our data indicate that mitochondria in KS keratinocytes are not only altered in structure, but also in their distribution and functionality.

## Discussion

The natural history of a genetic disease is not completely described by the simple identification of the causal mutation but requires also the understanding of biochemical and molecular mechanisms that are directly responsible of the phenotypic characteristics of the disease. Sometimes this is challenging given the multiple interactions of genes downstream of the mutation. This appears to be the case of KS for which several aspects of its pathogenesis remain uncertain. Since two of the unexplained features of KS, photosensitivity and cancer development, have been associated with oxidative stress, we have advanced the hypothesis that some clinical features of KS patients may be related to a redox imbalance and higher susceptibility to oxidative stress.

Our results cover mutation spectra and phenotype since they are based on three very different types of mutations. They include a simple hypomorphic mutation caused by a single amino acid substitution (SK3), a mutation resulting in a truncated protein (SK1), and a patient null for kindlin-1 (SK2). Furthermore, given the importance of age in the symptomatology of this disease, the three patients studied reflect different ages, two children (SK1 and SK2) and one adult (SK3). For redox studies, we also took into account the patients’ gender (all patients are females) and consequently, in every case the control was matched by age and gender. As for other genodermatoses, availability of samples to derive keratinocyte cultures is usually low. In spite of this drawback, our results, obtained with primary patient cells covering different mutations and ages, follow a clear trend regarding oxidative status and mitochondrial alterations.

The oxidative stress biomarker analysis revealed that GSSG/GSH ratio was higher in SK1 and SK3 keratinocytes (Figure 1). These results are linked to a lower capability of KS cells to synthesize GSH due to the down-regulation of the catalytic (GCLC) and the regulatory (GCLM) subunits of gamma-glutamyl cysteine ligase (GCL), the first rate limiting enzyme of GSH synthesis (Figure 1). Therefore, keratinocytes from KS patients are prone to oxidative stress likely leading to damage of different cellular components. Consistently, the lipoperoxidation product MDA showed high levels in KS keratinocytes. In addition, this difference was higher in cells from the adult patient (Figure 1). These results, which may reflect oxidative damage accumulation during ageing, are especially relevant in KS because MDA has been involved in the pathogenesis of skin alterations associated with non-melanoma and melanoma skin cancer [27] as well as photoaging [28]. Furthermore, MDA can bind to DNA producing mutagenic adducts [29]. MDA derivatives such as dihydropyridine (DHP)-type adducts including DHP-sysine [(S)-2-amino-6-(3,5-diformyl-4-methyl-4 h-pyridin-1-yl)-hexanoic acid] may accumulate under cellular redox unbalance in human tissues [30,31]. These products can originate phototoxicity as occurs in human retinal pigment epithelial cells [32] and could be major sensitizers of photooxidative stress in human skin cells [33].

Cytoplasmic and mitochondrial abnormalities in redox balance confirmed by Grx1-roGFP2 biosensors system, in KS keratinocytes (Figure 2) suggests that KS cells would not be competent to properly cope with an oxidative imbalance in these compartments.

Taken together, our results suggest that alterations of the redox balance in KS may be a potential explanation of the premature skin ageing and cancer prone phenotype of these patients.

As discussed above, our results with the biosensor probes point to the cytoplasm and the mitochondria as potential sources of ROS. For this reason, we decided to study morphological and functional alterations of the mitochondria of KS patients. Electron microscopy of both, skin biopsies and cultured keratinocytes from KS patients showed morphological alterations which are consistent with dysfunctional mitochondria (Figure 3) as previously described in UV-irradiated skin [34]. These results were confirmed by confocal microscopy which not only showed morphological alterations with the Mito Tracker probe but also functional abnormalities as revealed by the membrane potential-sensitive JC-1 dye (Figure 4). Similar results were reported with these two probes in studies of mitochondrial dysfunction in Parkinson's and Alzheimer's disease [35,36].

To our best knowledge, neither oxidative stress nor alterations in the mitochondria have been previously reported in KS. It is not yet clear whether a derangement of the redox status in KS cells is cause or consequence to mitochondrial dysfunction and morphological aberrations. The most likely explanation is that mitochondria are both the cause and effect of the oxidative stress. Therefore, we envisage a vicious circle where ROS-altered mitochondria results in increased mitochondrial oxidative stress.

At this point it is not clear how alterations in kindlin-1 may lead to generation of oxidative stress. It is unlikely that the pure disruption of the kindlin-1 synthesis would explain this phenomenon. A likely explanation is that alterations in kindlin-1 function lead to a disruption in the signal transduction pathways involving integrins and focal adhesions [37-40]. In fact, integrins modulate mitochondrial function by signalling through Rho GTPases leading to the increase in ROS formation. On the other hand, ROS play a role in the regulation of the early phase contact between integrins and extracellular matrix resulting in a positive feedback loop [39]. Not only mitochondrial ROS but also cytosolic ROS levels are affected by integrins, particularly in cooperation with growth factor stimulation [41,42]. Recent evidence from a conditional *Fermt1* knock out mouse model associated kindlin-1 deficiency to αv β6 integrin-mediated increase of TGFβ activation [40]. It has been shown that TGFβ-induced reduction of mitochondrial complex IV and respiration leads to increased ROS and decreased mitochondrial membrane potential associated with senescence in lung epithelial cells. TGFβ-1 induces prolonged mitochondrial ROS generation through decreased complex IV activity with senescent arrest in Mv1Lu cells [43]. It is thus tempting to speculate that these molecular events may be related to the oxidative stress and mitochondrial changes herein described. Further studies are, however, needed to establish whether TGFβ could be the link between deficient integrin signalling and oxidative stress.

## Conclusions

Our results suggest that KS keratinocytes are cells under severe oxidative stress, a condition which could underlie some of the obscure aspects of the disease such as photoaging, photosensitivity and ultimate, the high risk of cancer development.

## Additional files

Additional file 1:
**Table S1.** Age and sex description of the patients and controls used in this study. **Figure S1.** Expression analysis of Kindlin1 is KS keratinocytes. Western blot analysis was performed from control and KS keratinocytes. Cells from each patient show a different pattern of protein expression due to their different *FERMT1* mutations. α-tubulin was used as protein loading control. **Figure S2.** Patient specific redox biosensors analysis. The ratio 405/488 nm in control and KS keratinocytes from each patient infected either with retroviruses encoding Grx1-roGFP2 (a) or mito-Grx1-roGFP2 (b) are shown. Additional file 2: Figure S3. Quantification of JC-1staining by FACS analysis. Note mitochondrial depolarization in KS keratinocytes compared with their respective controls, as indicated by the decrease in the red/green fluorescence intensity ratio. Membrane potential reduction was statistically significant (*p < 0.05) after t-student test.

## Abbreviations

KS: Kindler Syndrome
ROS: Reactive Oxygen Species
MDA: Malondialdehyde
GSH: Glutathione
