# Supplementary material for: Oxidative stress and mitochondrial dysfunction in Kindler syndrome
Source: Orphanet J Rare Dis. 2014 Dec 21;9:211. doi: 10.1186/s13023-014-0211-8 (PMC4302591; doi:10.1186/s13023-014-0211-8)
Supplement: Additional file 1: — Table S1. Age and sex description of the patients and controls used in this study. Figure S1. Expression analysis of Kindlin1 is KS keratinocytes. Western blot analysis was performed from control and KS keratinocytes. Cells from each patient show a different pattern of protein expression due to their different FERMT1 mutations. α-tubulin was used as protein loading control. Figure S2. Patient specific redox biosensors analysis. The ratio 405/488 nm in control and KS keratinocytes from each patient infected either with retroviruses encoding Grx1-roGFP2 (a) or mito-Grx1-roGFP2 (b) are shown. [file 13023_2014_211_MOESM1_ESM.pdf]

Table S1

	Age (years)	Sex
Control 1 (HK C1)	5	Female
Control 2 (HK C2)	89	Female
Control 3 (HK C3)	17	Male
Patient 1 (SK1) (c.1371+4A>G)	4	Female
Patient 2 (SK2) (c.676dupC)	11	Female
Patient 3 (SK3) (c.1198T>C)	51	Female

Table S1: Age and sex description of the patients and controls used in this study.

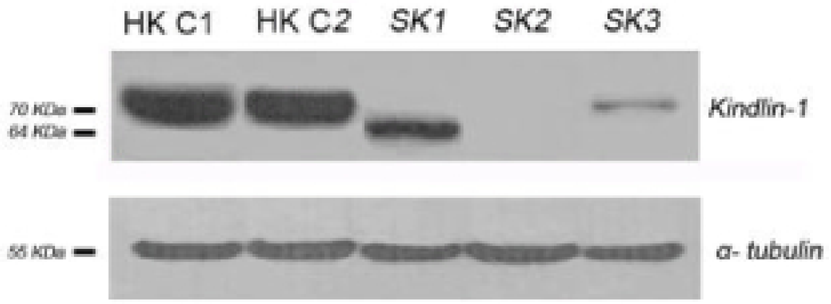

Figure S1

Figure S1: Expression analysis of Kindlin1 is KS keratinocytes. Western blot analysis was performed from control and KS keratinocytes. Cells from each patient show a different pattern of protein expression due to their different *FERMT1* mutations. α-tubulin was used as protein loading control.

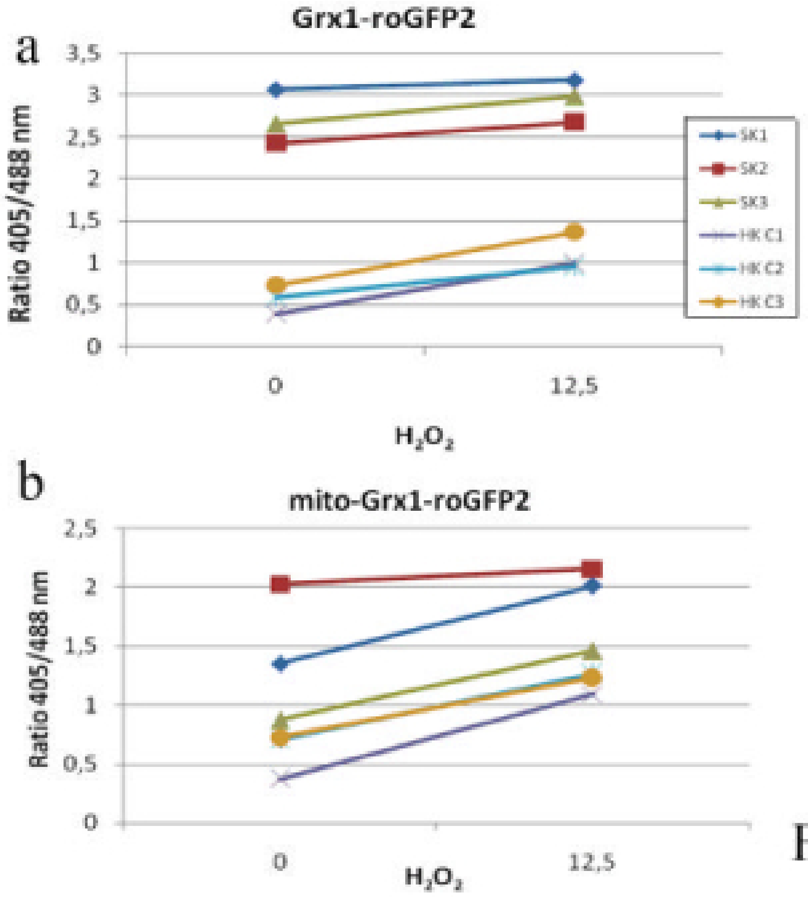

Figure S2

Figure S2: Patient specific redox biosensors analysis. The ratio 405/488 nm in control and KS keratinocytes from each patient infected either with retroviruses encoding Grx1-roGFP2 (a) or mito-Grx1-roGFP2 (b) are shown.
