# Supplementary material for: Oxidative stress and mitochondrial dysfunction in Kindler syndrome
Source: Orphanet J Rare Dis. 2014 Dec 21;9:211. doi: 10.1186/s13023-014-0211-8 (PMC4302591; doi:10.1186/s13023-014-0211-8)
Supplement: Additional file 2: Figure S3. — Quantification of JC-1staining by FACS analysis. Note mitochondrial depolarization in KS keratinocytes compared with their respective controls, as indicated by the decrease in the red/green fluorescence intensity ratio. Membrane potential reduction was statistically significant (*p < 0.05) after t-student test. [file 13023_2014_211_MOESM2_ESM.pdf]

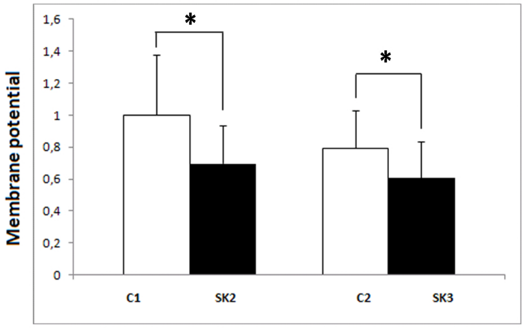

Figure S3

Figure S3: **Quantification of JC-1 staining by FACS analysis.** Note mitochondrial depolarization in KS keratinocytes compared with their respective controls, as indicated by the decrease in the red/green fluorescence intensity ratio. Membrane potential reduction was statistically significant (* $p<0.05$) after *t*-student test.
